# Emerging B and plasma cell-targeting immune therapies in idiopathic inflammatory myopathies

**DOI:** 10.3389/fimmu.2025.1581323

**Published:** 2025-07-17

**Authors:** Marwin Groener, Julie J. Paik

**Affiliations:** Division of Rheumatology, Department of Medicine, Johns Hopkins University School of Medicine, Baltimore, MD, United States

**Keywords:** idiopathic inflammatory myopathies (IIM), B cell targeted therapies, plasma cell targeting agents, CAR T, bispecific T cell engager (BiTE), myositis

## Abstract

Autoantibodies play a crucial role in the diagnosis and clinical characterization of idiopathic inflammatory myopathies (IIM). These antibodies are categorized into myositis-specific autoantibodies (MSAs), which are unique to IIM, and myositis-associated autoantibodies (MAAs), which can be seen with other autoimmune diseases. Both plasmablasts and plasma cells contribute to the production of these autoantibodies. Current B cell-targeted therapies, such as rituximab, have shown promise in refractory IIM, although their limitations – particularly in targeting plasmablasts and plasma cells – highlight the need for alternative agents with greater efficacy. This review discusses the evolving landscape of B cell and plasma cell-targeted immunotherapies in IIM, including next-generation anti-CD20 antibodies, BAFF inhibition, anti-CD19 CAR-T cells, BCMA-targeted therapies, and anti-CD38 antibodies. Most studies on the use of these novel treatment strategies in IIM have reported positive outcomes, although the number of patients treated is small. While these therapies represent a paradigm shift, further randomized clinical trials are needed to identify optimal strategies for IIM management and establish long-term safety and efficacy.

## Introduction

Myositis autoantibodies are a hallmark feature of idiopathic inflammatory myopathies (IIM), serving as both diagnostic markers and guide to clinical phenotypes. These autoantibodies are broadly classified into myositis-specific autoantibodies (MSAs), which are unique to IIM, and myositis-associated autoantibodies (MAAs), which may overlap with other autoimmune conditions. MSAs are associated with dermatomyositis (DM) (e.g. Anti-Mi-2, Anti-TIF1-γ, Anti-MDA5), anti-synthetase syndrome (AsyS) (e.g. anti-Jo-1, anti-PL-12, anti-EJ), and immune-mediated necrotizing myopathies (IMNM) (anti-SRP, anti-HMGCR). While direct pathogenicity is not confirmed for all MSAs, they are often linked to specific clinical manifestations, such as skin rashes, ILD, and variable muscle involvement. Anti-SRP and anti-HMGCR are reported to be pathogenic in mice and their titers correlate with disease activity (1–3).

**Figure 1:**
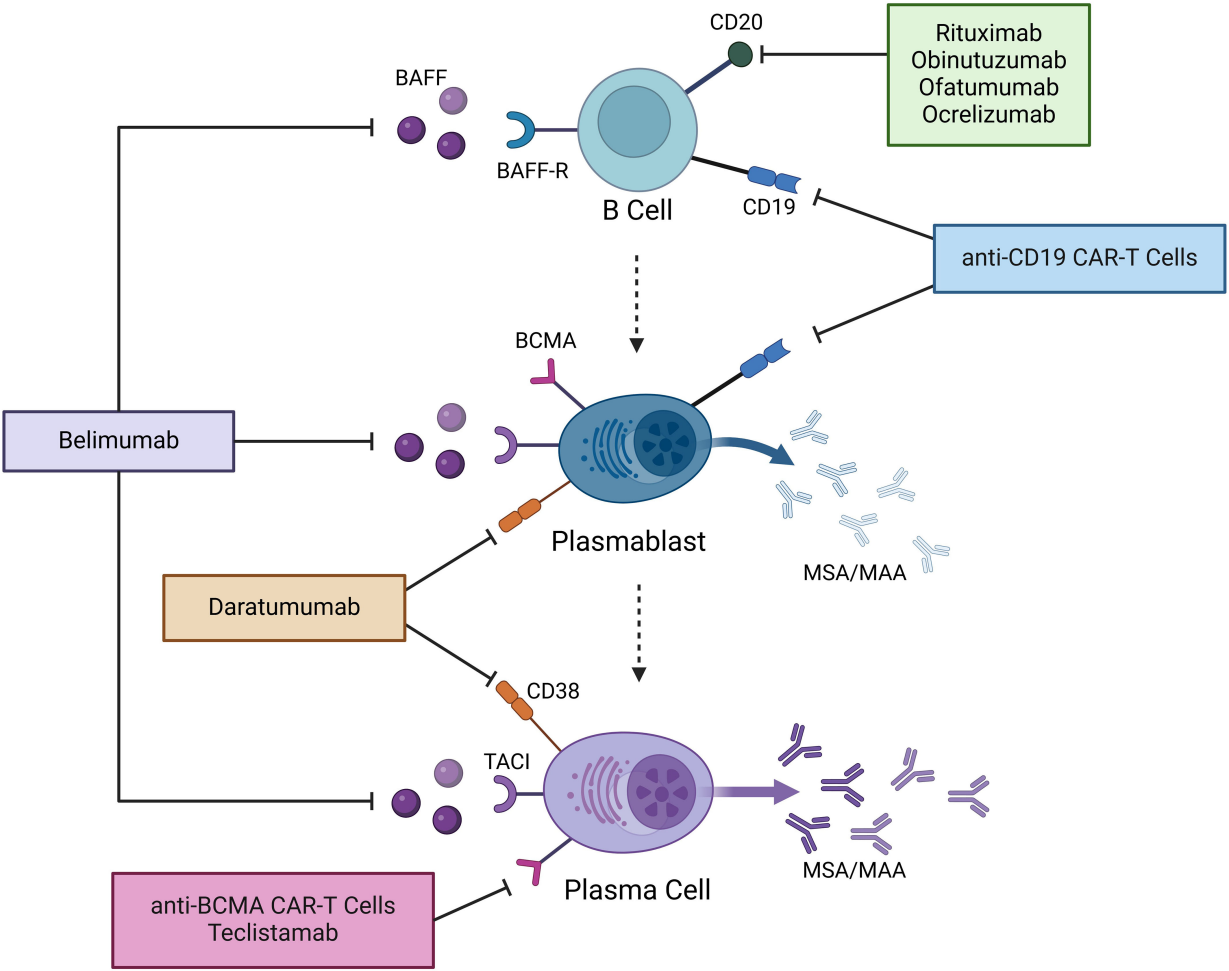
Mechanisms of action of novel B and plasma cell-targeted therapies in IIM. Therapies that have thus far been used in IIM are shown. BAFF, B cell activating factor; BAFF-R, B cell activating factor receptor; BCMA, B cell maturation antigen; TACI, Transmembrane Activator and CAML Interactor; MSA, myositis-specific autoantibodies; MAA, myositis-associated autoantibodies; CAR-T cells, chimeric antigen receptor T cells.

Understanding the key immune cells involved in autoantibody production is especially important in light of the many emerging B cell and plasma cell-targeted immune therapies, including CAR-T cells, among others, in IIM. B cell development begins with Pro-B cells in the bone marrow, which express both CD19 and CD38. They then develop into Pre-B cells and immature B cells, gaining additional expression of CD20. They migrate into the peripheral blood and lymph nodes as mature B cells, losing CD38 expression. Subsequently, they can then transform into memory B cells or antibody-producing cells. In general, antibodies are produced by two cell types: plasmablasts and plasma cells (PCs). Both arise from the B cell lineage after continued antigen stimulation from T cells in lymph nodes. Plasmablasts are part of the early humoral response, circulate in the blood, and have a life span of around 3 days (4). Notably, plasmablasts lose CD20 expression during maturation but retain CD19. PCs, on the other hand, mature from plasmablasts in the germinal centers of lymph nodes as a result of multiple cycles of high-affinity cloning. They are terminally differentiated and migrate to their survival niches in the spleen, bone marrow, and inflamed tissue, where they can be long-lived. They continuously produce antibodies that can be measured years after the initial antigenic stimulus, for example, a vaccine. Similarly, an antigenic stimulus is thought to trigger the production of autoantibodies by both plasmablasts and PCs in IIM (5, 6). PCs do not express CD19 or CD20. Instead, plasmablasts and PCs express B-cell maturation antigen (BCMA) and regain CD38. Therapies targeting these various B and plasma cell markers have been used in IIM, as outlined in **Figure 1**.

Given the suspected pathogenic role of myositis autoantibodies, there has been a keen interest in determining how they may play a direct role in the target muscle tissue. Recently, Pinal-Fernandez et al. demonstrated that there is autoantibody internalization in myositis (7). IgG immunofluorescence staining of myofibers from myositis patients corresponded with the respective autoantigen location of the serum MSA of each patient. For example, myofibers of patients with anti-HMGCR, anti-SRP, anti-MDA5, and anti-Jo-1 autoantibodies, which recognize cytoplasmic antigens, had cytoplasmic staining patterns; whereas, a nuclear pattern was observed in patients with anti-Mi2 antibodies, corresponding to the location of the Mi2/NuRD complex in the nucleus.

With increasing literature supporting the possible direct pathogenic role of myositis autoantibodies in IIM, it is not surprising that B-cell targeted therapies, such as the anti-CD20 monoclonal antibody rituximab, have been explored in treating IIM. While the 2013 Phase 3 Rituximab in Myositis (RIM) trial did not meet its primary endpoint, it is important to note that 83% of patients met the definition of improvement and autoantibody positivity predicted a better response (8, 9). Furthermore, a decrease in autoantibody levels was associated with clinical improvement (10). A more recent study also reported the efficacy of rituximab in a case series of 9 refractory anti-HMGCR patients (11). In a Korean cohort of 16 refractory IIM patients with a median of 3.6 prior therapies that were treated with rituximab, 25% had complete remission and 50% had partial remission at a median of 24 weeks of follow-up (12).

Our current first-line therapies for IIM include glucocorticoids and disease-modifying antirheumatic drugs (DMARDs), such as methotrexate, azathioprine, and mycophenolate. IVIG was FDA-approved to treat dermatomyositis in July 2021 and is used as a second-line treatment (13). Similarly, Rituximab is often used as a second-line agent. Novel B cell and plasma cell targeting immunotherapies have entered the autoimmune treatment landscape in the past several years. The purpose of this review is to summarize the current evidence of the use of these agents in IIM and highlight the potentially pivotal role in changing the treatment paradigm.

## Emerging B cell and plasma cell-targeting immunotherapies

Given the number of B-cell and plasma cell-targeting immunotherapies, we will discuss these by organizing our review into the key targets of the following therapeutic agents (see **Figure 1**).

### Medications targeting CD20: next-generation anti-CD20 monoclonal antibodies

Next-generation anti-CD20 antibodies have been designed to have better complement-dependent and antibody-dependent cytotoxicity than rituximab. They were also designed with the goal of lower immunogenicity leading to longer efficacy and fewer medication reactions (14). **Supplementary Table S1** offers an overview of anti-CD20 antibodies that have been used in IIM (14–16).

Obinutuzumab is a glycosylated humanized anti-CD20 antibody that is currently FDA-approved for the treatment of follicular lymphoma and chronic lymphocytic leukemia (CLL). It was used in a series of five patients with treatment-resistant IIM (2 ASyS, 1 DM, 1 overlap myositis, 1 SLE/DM overlap) which was presented as an abstract at the 2024 American College of Rheumatology annual scientific meeting (17). Four patients were allergic to rituximab and one had failed previous therapy with rituximab. Peripheral CD19-positive B cells were undetectable up to one year after obinutuzumab therapy. Clinical improvement was noted in skin, muscle, and joint disease. Lung imaging and PFTs remained stable, despite symptomatic respiratory improvement. There were no significant adverse events. Another patient with refractory anti-Jo-1 ASyS had improvement of myalgias, strength, and creatine kinase (CK) levels after one cycle of obinutuzumab (18).

Ofatumumab is a fully human anti-CD20 antibody used for the treatment of multiple sclerosis and CLL that is administered subcutaneously. Two case reports from China describe the use of ofatumumab in refractory anti-SRP IMNM. The first patient had significant muscle weakness that did not improve with steroids, tacrolimus, and IVIG (19). After starting treatment with 20mg of ofatumumab given as three doses spread over five months, she gradually regained her strength over six months following the first dose with a simultaneous decrease in CK. The second patient had severe muscle involvement with dysphagia and dyspnea despite steroids and IVIG (20). She had marked improvement in strength only one month after the first dose of 20mg ofatumumab, which was given for a total of three monthly doses. No significant side effects or infections were noted in either case.

Ocrelizumab is a humanized anti-CD20 antibody that is FDA-approved for multiple sclerosis. It was used in a patient with refractory ASyS but did not have any efficacy, thus leading to follow-up therapy with anti-CD19 CAR-T cells (21) - this case is discussed further below. To our knowledge, the use of other next-generation anti-CD20 monoclonal antibodies in IIM, such as ublituximab and veltuzumab, has not been reported in the literature.

### Medications targeting B cell activating factor

B cell activating factor (BAFF), also known as B-lymphocyte stimulator (BLyS), is a cytokine critical for B cell survival, differentiation, and function. BAFF binds to three receptors: BAFF-R, TACI (Transmembrane Activator and CAML Interactor), and to a much weaker degree BCMA (B cell maturation antigen) (22), each playing distinct roles in B cell regulation. It supports the survival of plasmablasts and PCs through TACI-mediated signaling, thereby sustaining autoantibody production. BAFF levels are increased in patients with IIM and they correlate with disease activity (23–25). Furthermore, BAFF and BAFF receptors are increased in the muscle tissue of patients with DM compared to controls (26).

Belimumab, a fully human monoclonal antibody binding soluble BAFF, is currently FDA-approved for systemic lupus erythematosus. There are several studies on belimumab in IIM, reporting the highest number of patients that have thus far been treated with novel B or plasma cell-targeted agents (**Table 1**). A 40-week randomized, double-blind, placebo-controlled trial of belimumab versus placebo was conducted in 15 patients with refractory IIM (27). Patients were allowed to continue their background therapy - most commonly one background immunosuppressant with additional prednisone or IVIG. Belimumab was given at a dose of 10m/kg IV every two weeks for the first three doses, then every 4 weeks. More patients in the belimumab arm reached a total improvement score (TIS) of ≥40 and achieved the definition of improvement (DOI), however, the differences were not statistically significant. Notably, there was no improvement in the placebo arm between weeks 40 and 64 after switching to the open-label extension. The results were limited by the relatively small number of patients and high placebo response.

**Table 1:** Novel B and plasma cell-targeted therapies in IIM.

Target	Therapy	IIM-Subtype	Number of Treated Patients	MSA/MAA	Clinical Features	Outcomes	Add-on Therapies	References
**CD20**	Obinutuzumab	ASyS*	3	U1-RNP, PL-12, Jo-1, SSA, Ro-52	Muscle, joint, lung	Improvement in muscle, joint	GC, MTX, AZA	(17, 18)
		DM/OM	3	RNP, Sm, dsDNA, SSA, Ro-52, EJ	Muscle, skin, joint, lung	Improvement in muscle, skin, joint	GC, MTX	(17)
	Ofatumumab	IMNM	2	SRP, Ro52	Muscle	Improvement in muscle	GC, IVIG	(19, 20)
	Ocrelizumab	ASyS	1	Jo-1, PM-Scl, Ro52	Muscle, skin, joint, lung	No improvement	GC	(21)
**CD19**	Autologous CAR-T Cells	ASyS	4	Jo-1, PM-Scl, PL-7, Ro52	Muscle, skin, joint, lung	Improvement in all domains. One relapse; One add-on of DMARD after CAR-T	MMF, AZA	(21, 31, 34)
		jDM	1	None identified	Skin, muscle	Drug-free remission	No	(35)
		IMNM	1	SRP, Ro52	Muscle	Drug-free remission	No	(36)
	Allogeneic CAR-T Cells	IMNM	1	SRP	Muscle	Drug-free remission	No	(37)
**BCMA**	Autologous CAR-T Cells	IMNM	1	SRP, SSA, Ro52	Muscle, ocular sicca	Drug-free remission	No	(42)
		ASyS	1	Jo-1	Muscle, joint, lung	Drug-free remission	No	(33)
	Teclistamab (BiTE)	DM	1	MDA5	Muscle, skin, joint, lung	Improvement in all domains	No	(41)
**BAFF**	Belimumab	DM, PM, IMNM	10	Jo-1, SSA, SRP, HMGCR	Muscle, skin	Global improvement – but no benefit over placebo.	GC, IVIG, DMARD	(27)
		IMNM	1	SRP	Muscle	Improvement in muscle	GC, DMARD	(28)
		DM, ASyS jDM	13	NXP2, MDA5, Jo1, KS, RNP, Ro52, other	Muscle, skin, joint, lung	Improvement in all domains	GC, DMARD, IVIG	(29)
**CD38**	Daratumumab	DM	3	MDA5, Ro52	Muscle, skin, joint, lung	Improvement in all domains	GC, IVIG, DMARD, JAKi	(45–47)
		IMNM	1	SRP	Muscle	Improvement in muscle	GC, IVIG, DMARD	(48)
		ASyS	2	Jo-1, Ro52	Muscle, joint, lung	No response	GC, DMARD	(33, 49)

Overview of novel B and plasma cell targeted therapies in IIM with IIM-subtypes, number of treated patients, autoantibodies, organ involvement, outcomes, and concomitant immunosuppression if applicable. *Asterisk: IIM-subtypes were pooled if possible with available data. ASyS: anti-synthetase syndrome; DM: dermatomyositis; OM: Overlap-myositis; IMNM: immune-mediated necrotizing myositis; jDM: juvenile dermatomyositis; BiTE: bispecific T cell-engaging antibody; GC: glucocorticoids; MTX: methotrexate; AZA: azathioprine; IVIG: intravenous immunoglobulin; MMF: mycophenolate mofetil; DMARD: disease-modifying antirheumatic drugs; JAKi: Janus kinase inhibitor.

A case report describes a patient with anti-SRP IMNM who had recurrent disease flares despite treatment with steroids, methotrexate, and cyclosporine (28). Belimumab was added at a dose of 10 mg/kg once every two weeks for six weeks, followed by 10 mg/kg once a month, leading to clinical improvement and normalization of CK levels over the next 23 weeks.

In a retrospective study of 13 patients with refractory adult or juvenile DM who were treated with belimumab, all but one patient reached the primary endpoint of a decrease in Myositis Intention-to-Treat Activity Index scores by at least 20% at week 12 (29). Most patients remained on at least one background immunosuppressant and steroids throughout the treatment period. Improvement in both muscle and skin disease was seen. The patients remained clinically stable at 24 weeks without any observed severe adverse events.

### Medications targeting CD19

CD19-targeting therapies have recently shown significant promise in autoimmune disease. This includes anti-CD19 chimeric antigen receptor T cells (CAR-T cells) and anti-CD19 bispecific T-cell engaging antibodies (BiTE). CD19 has a broader range of expression along the B cell lineage than CD20. It is additionally expressed by pro-B cells and plasmablasts but is absent from long-lived plasma cells (30).

CAR-T cells are genetically modified T cells that express a chimeric antigen receptor for a specific target (e.g. CD19). The T cells can be derived from the patient or a donor, referred to as autologous and allogeneic, respectively. After infusion of the CAR-T product, usually preceded by lymphodepletion through chemotherapy, the CAR-T cells expand *in vivo* and attack all cells expressing the desired target, leading to deep depletion of these cells in both circulation and tissue (30). Given the rapid evolution of the field and the large number of ongoing clinical trials, we are highlighting published reports of CAR-T cell therapy in IIM but acknowledge that there will likely be more cases by the time our review is published.

Autologous anti-CD19 CAR-T cells were successfully used by the group in Erlangen, Germany, in three patients with ASyS (21, 31, 32). The first patient was Jo-1 positive, the second patient was Jo-1, PM-Scl, and Ro52 positive, and the third patient was PL-7 and Ro52 positive. All three patients were recalcitrant to multiple lines of therapy including IVIG and rituximab, one patient had additionally received ocrelizumab without improvement of her disease. After treatment with anti-CD19 CAR-T therapy, all patients met ACR-EULAR Major Clinical Response Criteria with normalization of manual muscle testing 8 (MMT-8) scores and CK within 3 months. CAR-T treatment was relatively well tolerated with cytokine release syndrome (CRS) grade 1 and 2, and immune effector cell‐associated neurotoxicity syndrome (ICANS) grade 1. Interestingly, Jo-1 antibody levels significantly dropped in one patient but remained stable in another. PL-7 and PM-Scl100 antibody levels remained relatively unchanged. This underscores multiple hypotheses: 1) autoantibodies that do not diminish after anti-CD19 CAR-T therapy are likely produced by CD19-negative PCs; 2) the same autoantibody (here Jo-1) can be produced by CD19-positive plasmablasts and CD19-negative PCs to different degrees in different patients; 3) Jo-1, PL-7, and PM-Scl100 antibody levels do not seem to correlate with disease activity in these three patients. Time will tell if detectable autoantibody levels after anti-CD19 CAR-T therapy are associated with a higher risk of disease relapse in IIM patients. Indeed, a follow-up report was published about the patient with anti-Jo-1 ASyS who had elevated antibody titers after CD19-CAR-T therapy (33). After 9 months of drug-free remission, the patient experienced gradually worsening weakness and rising CK, without recurrence of ILD. A second cycle of the same anti-CD19 CAR-T product was administered after repeat lymphodepletion with cyclophosphamide and fludarabine. No CAR-T expansion and B cell depletion was observed. T-cell-mediated reactivity against the CAR-T cells was suspected. Subsequent treatment with daratumumab, an anti-CD38 monoclonal antibody targeting PCs, resulted in improvement in muscle strength and CK levels, without a meaningful decrease in Jo-1 antibody titers. However, muscular symptoms recurred after 5 months. Anti-BCMA CAR-T cell therapy was chosen as the next step with the goal of deeper depletion of plasma cells. This resulted in clinical remission of her muscular disease after 3 weeks. The response was maintained 9 months post-therapy. Interestingly, Jo-1 antibody titers decreased markedly after anti-BCMA CAR-T therapy, but did not turn negative. This case shows that lymphodepletion with cyclophosphamide and fludarabine alone without CAR-T expansion is not sufficient to induce disease remission. Furthermore, it highlights that sparing the PC compartment with anti-CD19 therapy can result in disease recurrence, possibly due to persistent autoantibody production. Additional reports on the use of anti-BCMA and anti-CD38 therapy in IIM are detailed further below.

Another case of anti-CD19 CAR-T therapy in IIM describes a patient with refractory anti-Jo-1 ASyS who had been treated with multiple agents including IVIG, rituximab, and baricitinib (34). The patient had progression of disease with worsening dyspnea, muscle pain, and muscle weakness. After treatment with autologous anti-CD19 CAR-T cells, she initially had an increase in CK levels and clinical disease activity, despite undetectable peripheral B cells. An increase in (non–CAR) terminal effector memory T cells (CD45RA+CD27-) among CD8-positive T cells was noted, so she was started on T cell targeting therapy first with azathioprine, then mycophenolate around one month after CAR-T infusion. Clinical disease activity and CK levels subsequently improved with a slow decline of her Jo-1 antibody levels over the ensuing 7 months. This instructive case highlights the complicated relationship between T cells and B cells in IIM and how in select patients, treatment needs to be guided by the precise understanding of a patient’s inflammatory milieu.

A 12-year-old boy with juvenile dermatomyositis had refractory disease despite treatment with various lines of therapy including methotrexate, rituximab, cyclophosphamide, steroids, and IVIG (35). He did not have identifiable MSA or MAA, despite positive anti-nuclear antibody testing. He responded well to treatment with autologous anti-CD19 CAR-T cells with normalization of muscle strength and near resolution of skin disease. While his CK levels were normal even before therapy, his muscle involvement had resolved on a follow-up muscle MRI 6 months after CAR-T therapy.

Two patients with anti-SRP IMNM responded well to treatment with anti-CD19 CAR-T cells. One patient was treated with an autologous product leading to a reduction in CK levels and improvement of his MMT-8 over the following 16 weeks (36). A decrease between 54-81% in his autoantibody levels (anti-SRP isotypes, anti-Ro52) was observed without a change in vaccine-associated antibody levels. This was attributed to the production of autoantibodies by plasmablasts and vaccine-antibodies by PCs. Treatment was well-tolerated without CRS or ICANS. Another patient with anti-SRP IMNM was treated with an allogeneic anti-CD19 CAR-T product (37). She had severe muscle involvement despite treatment with steroids, IVIG, and multiple other agents including cyclophosphamide, but not rituximab. After lymphodepletion and CAR-T infusion, her disease rapidly improved and met major response as defined by the ACR/EULAR Total Improvement Score (TIS) of 100 after two months. SRP antibody levels were undetectable after one month. She tolerated the treatment well without signs of CRS or graft-versus-host disease.

There are additional therapies targeting CD19 that have not yet been used in IIM. The anti-CD19 BiTE blinatumomab binds CD3 with one arm and CD19 with the other, leading to the destruction of CD19-positive cells by the patient’s own T cells. It was successfully used in patients with refractory rheumatoid arthritis and systemic sclerosis (38, 39). Inebilizumab, a monoclonal antibody targeting CD19, is FDA-approved for Neuromyelitis Optica Spectrum Disorder and has recently shown promise in the treatment of IgG4-related disease (40).

### Medications targeting B cell maturation antigen

B cell maturation antigen (BCMA) is a transmembrane receptor expressed mainly on late-stage memory B cells and to a higher degree on plasmablasts and PCs. Its main ligand is APRIL, leading to downstream signaling that promotes plasmablast and PC survival. It is absent in earlier stages of the B cell lineage, making it a promising target for plasmablast and plasma cell-directed therapy.

Recently, the anti-BCMA bispecific T-cell engager teclistamab was used in several patients with refractory autoimmune disease, including an anti-MDA5 positive patient with dermatomyositis (41). This patient had joint, skin, muscle, and lung involvement that was refractory to multiple immunosuppressive agents, including methotrexate, azathioprine, mycophenolate, hydroxychloroquine, a JAK inhibitor, rituximab, steroids, and IVIG. After treatment with teclistamab, she had significant improvement in all disease domains at 12-week follow-up with a decrease in her Cutaneous Dermatomyositis Disease Area and Severity Index (CDASI) and Disease Activity Score 28 for Rheumatoid Arthritis with CRP (DAS28-CRP), as well as an increase in her lung diffusion capacity and strength by MMT-8. No CRS, ICANS, or infections were noted, although predictably hypogammaglobulinemia occurred with targeting of the entire PC compartment. Anti-MDA5 titers significantly decreased but remained detectable on follow-up. The benefits of BiTE over CAR-T therapy include higher scalability, no need for cell apheresis or lymphodepletion, and no obvious risk of secondary T cell malignancy.

Another case describes a 25-year-old man with refractory anti-SRP IMNM with Sjögren’s disease overlap who was treated with an autologous anti-BCMA CAR-T product (42). Over the seven years of his disease, he had received several courses of therapy including IVIG, rituximab, cyclophosphamide, and plasmapheresis, but continued to have disease flares. He was bed-bound with CK levels of > 4000 and persistently elevated SRP antibody titers. After lymphodepletion and CAR-T infusion, the patient had undetectable Ro52 and SSA antibody levels with a significant decrease in SRP antibody levels by 3 months of follow-up. He had progressive clinical improvement of his muscle strength and CK levels with near-normal exam by 9 months post-treatment and remained in long-term remission after 18 months. Side effects were grade 1 CRS and hypogammaglobulinemia.

Lastly, treatment with anti-BCMA CAR-T therapy was successful in an anti-Jo-1 ASyS patient who experienced recurrence of myositis after anti-CD19 CAR-T therapy. She subsequently did not respond to repeat anti-CD19 CAR-T cells and only transiently responded to daratumumab. This case is detailed in the section on anti-CD19 therapies (33).

It is important to note that anti-BCMA CAR-T therapy leads to a decrease in vaccine-associated humoral immunity as compared to anti-CD19 CAR-T therapy due to the depletion of plasma cells, which are essential for sustained antibody production (43).

### Medications targeting CD38

CD38 is a transmembrane glycoprotein that is highly expressed on plasmablasts and plasma cells, making it a target for patients with multiple myeloma. It is also expressed by other cell types including natural killer cells, plasmacytoid dendritic cells, and memory T cells (44). Daratumumab is an FDA-approved monoclonal antibody for the treatment of multiple myeloma. It has been used successfully in several different autoimmune diseases, including four cases of refractory IIM (45–48). Three patients had anti-MDA5 DM, all with significant lung involvement (45–47), the other patient had severe anti-SRP IMNM with dysphagia and diaphragmatic involvement (48). All patients had been treated with steroids, IVIG, B cell depletion with rituximab or cyclophosphamide, and other therapies. Despite this, they had ongoing disease activity with detectable autoantibodies, suggesting persistent autoreactive plasma cells. Treatment with daratumumab in addition to background therapy led to clinical improvement in all four patients with a reduction of autoantibody titers over the course of one to four months. The dosing was either 1800mg subcutaneous or 16mg/kg IV once weekly, given for a minimum of four weeks in each patient. The main complications noted were bacterial infections, especially in those requiring ICU care, and hypogammaglobulinemia.

It is important to note two reports of unsuccessful use of daratumumab in IIM. As mentioned earlier, an anti-Jo-1 ASyS patient relapsed 9 months after anti-CD19 CAR-T therapy. Repeat anti-CD19 CAR-T cells did not expand. Treatment with bimonthly subcutaneous daratumumab at 1800mg with concomitant dexamethasone led to a transient improvement in muscle symptoms and CK, without significant reduction in anti-Jo-1 titers. The patient had recurrence of her myositis with CK elevation and weakness after 5 months. Drug-free remission was achieved with subsequent anti-BCMA CAR-T therapy (33). A second patient, a 25-year-old male with refractory anti-Jo-1 ASyS manifested by ongoing muscle weakness with elevated CK and ILD had ongoing disease progression despite high-dose steroids of up to 0.5mg/kg/day and multiple immunosuppressants (49). He was treated with five 4-week cycles of weekly 1800mg daratumumab. Despite this, there was no improvement of his disease and prednisone could not be tapered below 30mg daily. He later died from SARS-CoV-1 pneumonia. Levels of Jo-1 antibody titers throughout treatment were not reported. Other anti-CD38 antibodies, such as felzartamab or isatuximab, have not been reported to be used in the treatment of IIM.

## Discussion

Emerging immunotherapies to target B cells and plasma cells more effectively in autoimmune diseases have garnered significant attention in recent years.

The treatment of IIM thus far has relied on using corticosteroids and immunosuppressants such as mycophenolate mofetil, azathioprine, or methotrexate first line. A step-up approach of adding additional B cell depleting agents has traditionally included rituximab, but not all patients respond to these agents. Emerging immunotherapies originally adopted from oncology have recently entered the autoimmune treatment landscape, including myositis, yielding striking improvements in disease activity. For example, treatment with anti-CD19 and anti-BCMA CAR-T cells led to dramatic improvement in previously refractory patients. Targeting BAFF with the monoclonal antibody belimumab has shown promise, although the Phase 2 clinical trial in refractory IIM did not meet its primary endpoint. Daratumumab, an anti-CD38 monoclonal antibody, was also successful in a few cases of IIM who did not respond to previous B cell depletion, highlighting a potential role in patients with plasma cell-driven disease.

The scientific community has recently taken a keen interest in CAR-T cell therapy for autoimmune disease, spurred by highly successful initial reports. While this novel therapeutic approach seems promising, several limitations need to be acknowledged. Essentially all initial patients were treated under compassionate use and not in the form of a controlled clinical trial, which introduces publication bias for successful outcomes and limits real-world applicability. Furthermore, CAR-T cell therapy comes with the risk of significant short- and long-term adverse effects. In most cases, conditioning with cyclophosphamide and fludarabine is necessary to deplete host lymphocytes and allow for adequate CAR-T cell expansion. This leaves patients vulnerable to bacterial, fungal, and viral infections, particularly in the first month after treatment (50). Additionally, CRS and ICANS are common in the first days after CAR-T infusion and currently require inpatient monitoring to intervene appropriately. Long-term risks include infection until B cell reconstitution and loss of vaccine memory with the depletion of plasma cells in anti-BCMA CAR-T therapy. There is also a small risk of secondary CAR-T malignancies, mostly lymphomas, which would be devastating if they arise in a patient with otherwise treatable autoimmune disease (51). Larger clinical trials are needed to determine long-term efficacy, and short- and long-term safety for CAR-T cell therapy in our patient population.

As with anti-BCMA therapy, plasma cell depletion with anti-CD38 antibodies carries the risk of hypogammaglobulinemia and loss of humoral memory, which can have an important impact on vaccine efficacy in our already immunosuppressed patients.

The reports on these novel agents in IIM offer several interpretations for their future use. Using anti-CD20 therapy with rituximab or next-generation antibodies should be explored as a first-line therapy in IIM in future clinical trials. While anti-BAFF therapy did not outperform placebo in the discussed randomized controlled trial, it could potentially be used for long-term maintenance to prevent B and plasma cell-repopulation after initial depletion. This strategy was recently successful in achieving long-term remission for three years in two patients with lupus nephritis after induction therapy with daratumumab (52). Plasma cell-targeted therapy with anti-CD38 or anti-BCMA treatments could prove useful as a second-line therapy in IIM patients who have active disease and persistently elevated autoantibodies despite peripheral B cell depletion. Similarly, CAR-T cell and BiTE therapy can be reserved for severe refractory cases. BiTE, simple antibody infusions, could potentially be used on a larger scale than CAR-T cell therapy while mitigating the malignancy risk.

Further research is needed to determine the role of individual MSA/MAAs in IIM pathogenesis. While HMGCR and SRP antibody titers correlate with disease activity and are pathogenic in mice, this is less clear for other MSAs. The highlighted reports on plasmablasts and PC-targeting therapies give us a few further insights. Several antibody titers decreased or even normalized after anti-CD19 CAR-T therapy, including SRP, Jo-1, and Ro52. However, one patient with unchanged anti-Jo-1 titers after CAR-T therapy still had drug-free remission for several months before experiencing disease relapse. Jo-1 antibodies might not be directly pathogenic, but elevated titers could indicate the potential for disease relapse. The treatment effect of B and plasma cell-depleting therapies is likely also mediated through the loss of cell-mediated immunity, rather than just a decrease of autoantibodies alone. It seems that antibodies are produced by both plasmablasts and PCs to various degrees. Experience from lupus patients after anti-CD19 CAR-T therapy demonstrates a marked decrease in anti-histone and anti-dsDNA titers, while Ro60 remains unchanged. It is thus likely that Ro60 antibodies are preferentially produced by long-lived CD19-negative PCs (53). PC-targeted therapy with anti-CD38 or anti-BCMA agents led to a marked decrease in all antibody titers including MDA5, SRP, Jo-1, and SSA in our reported cases. At this time, the optimal target antigen for an individual patient remains unclear. PC-depleting therapies appear more effective at decreasing global autoantibodies, but this must be balanced against the increased infection risk that comes with eliminating the humoral memory.

While the advent of B and plasma cell-targeted therapies in IIM is a significant development in rare diseases, caution is needed given the side effects of these medications and challenges of trial recruitment in IIM. The explosion of clinical trials in IIM in the past several years has brought significant challenges to recruitment, especially with a long placebo arm in Phase 3 trials (54). As these new therapies evolve, future clinical trials will require meticulous planning and thoughtful trial design. For example, employing innovative trial designs such as adaptive or platform clinical trials can facilitate recruitment, efficiency, and ultimately aim for better patient outcomes.

In conclusion, the evolving landscape of B cell and plasma cell-targeted therapies in IIM holds unprecedented promise, offering the hope of sustained remission. While we must proceed with caution to ensure the safety of our patients, these new treatments may also dictate a paradigm shift in the treatment of IIM and many other autoimmune diseases.
