# Supplementary material for: Emerging B and plasma cell-targeting immune therapies in idiopathic inflammatory myopathies
Source: Front Immunol. 2025 Jul 17;16:1581323. doi: 10.3389/fimmu.2025.1581323 (PMC12310725; doi:10.3389/fimmu.2025.1581323)
Supplement: Supplementary file 1 [file Table1.docx]

| Antibody | Rituximab | Obinutuzumab | Ofatumumab | Ocrelizumab |
| --- | --- | --- | --- | --- |
| Structure | Chimeric | Glycoengineered Humanized | Fully human | Humanized |
| Route | IV | IV | SQ | IV |
| Primary MOA | CDC > ADCC | ADCC > CDC | CDC > ADCC | ADCC > CDC |
| Side Effects | Infusion reactions, infections, hypogammaglobulinemia, neutropenia | | | |

**Supplementary Table 1. Overview of anti-CD20 monoclonal antibodies with reported use in IIM**

**Supplementary Table 1. Overview of anti-CD20 monoclonal antibodies with reported use in IIM.** IV: Intravenous; SQ: subcutaneous; CDC: complement-dependent cytotoxicity; ADCC: antibody-dependent cellular cytotoxicity
